# “Circulating Tumor Cells: Finding Rare Events for a Huge Knowledge of Cancer Dissemination”

**DOI:** 10.3390/cells9030661

**Published:** 2020-03-09

**Authors:** Catherine Alix-Panabières

**Affiliations:** Laboratory of Rare Human Circulating Cells (LCCRH), University Medical Centre of Montpellier, 641 Avenue du Doyen Gaston Giraud, 34093 Montpellier CEDEX 5, France; c-panabieres@chu-montpellier.fr; Tel.: +33-(0)4-1175-9931; Fax: +33-(0)4-1175-9933

Circulating tumor cells (CTCs) as real-time liquid biopsy [1] can be used to obtain new insights into the biology of the metastatic cascade, and as a companion diagnostic to improve the stratification of therapies and to obtain new insights into therapy-induced selection of cancer cells. Combining different circulating biomarkers, such as CTCs, circulating tumor DNA (ctDNA) and extracellular vesicles (EVs) analysis, will provide different and complementary information. Technical and clinical assay validation in big cohorts of cancer patients is crucial and can be achieved in international consortia such as the European Liquid Biopsy Society (ELBS) [2].

This Special Issue, “Circulating Tumor Cells: Finding Rare Events for A Huge Knowledge of Cancer Dissemination”, includes 23 articles written by experts in this field and covers multiple facets of CTCs in order to assemble a huge corpus of knowledge on cancer biology with emphasis on (i) technical challenges to enrich, detect, isolate and characterize CTCs at the single cell level, (ii) cancer biology with emphasis on metastasis including cancer stemness, epithelial-mesenchymal transition as well as immunomodulation of tumor cells, (iii) clinical studies on liquid biopsy, a new diagnostic concept introduced and coined for the first time in 2010 [1] for the analysis of CTCs and now extended to material (in particular DNA and EVs) released by tumor cells in the peripheral blood of cancer patients (Figure 1).

TECHNICAL CHALLENGES. Efficient enrichment of CTCs can be achieved by approaches that exploit differences between tumour cells and blood cells. Subsequently, enriched CTC populations might still contain among them hundreds to thousands of undesirable leukocytes, which requires the use of reliable methods to identify CTCs. Rossi et al. also highlighted the two major issues in the CTC field: rarity and heterogeneity [3]. Most current CTC assays use the same identification step as the FDA-approved CellSearch^®^ system. However, using physical properties to enrich CTCs, Obermayr et al. showed that CTCs enriched by the Parsortix system in small-cell lung cancer patients can be assessed using epithelial and neuroendocrine cell lineage markers at the molecular level [4]. Moreover, Bailey et al. reported emerging technologies for the capture of CTC/microemboli showing important biological and functional information that can lead to important alterations in how therapies are administered [5]. Moreover, Weerakoon-Ratnayake et al. detailed a novel microfluidic technology (i.e., microtrap device) that can perform immunophenotyping and FISH on CTCs [6].

One of the greatest challenges in neuro-oncology is the theranostic of leptomeningeal metastasis, brain metastasis and brain tumors, which are associated with poor prognosis in patients. Although extracranial metastases in glioma patients are rarely observed, recent studies have shown the presence of CTCs in the bloodstream. Bang-Christensen et al. demonstrated how the recombinant malaria VAR2CSA protein (rVAR2) can be used for the capture and detection of glioma cell lines and identified a panel of proteoglycans, known to be essential for glioma progression [7]. Cerebrospinal fluid (CSF) is one of the promising diagnostic targets because CSF passes through the central nervous system, harvests tumor-related markers from brain tissue and then, delivers them into peripheral parts of the human body where CSF can be sampled using minimally invasive and routine clinical procedure. Sindeeva et al. outlined the advantages, limitations and clinical utility of emerging liquid biopsy in vitro and photoacoustic flow cytometry (PAFC) in vivo for assessment of CSF markers including CTCs, ctDNA, miRNA, proteins, exosomes and emboli [8]. 

Chernysheva et al. studied disseminated tumor cells (DTCs) as a prognostic factor in many non-hematopoietic tumors [9]. They evaluated the possibility of detecting subsets of melanoma DTCs in the bone marrow based on the expression of a cytoplasmic premelanocytic glycoprotein HMB-45 using flow cytometry.

In addition to CTCs, circulating EVs can be of important interest. For instance, large tumor-derived extracellular vesicles (tdEVs) detected in blood of metastatic prostate, breast, colorectal, and non-small cell lung cancer patients are negatively associated with the overall survival of patients. Nanou et al. investigated whether, similarly to tdEVs, leukocyte-derived EVs (ldEVs) could also be detected in EpCAM-enriched blood [10].

CANCER BIOLOGY. The huge advantage of CTCs against other blood biomarkers is that the knowledge derived from a single-cell analysis of CTCs can be obtained at the DNA, RNA, and protein level [11].

As a first example, the expression of the androgen receptor splice variant 7 (ARV7) in circulating tumor cells (CTCs) has been associated with resistance towards novel androgen receptor (AR)-targeting therapies. A highly sensitive and specific qPCR-based assay was developed by Hille et al., allowing detection of ARV7 and keratin 19 transcripts from as low as a single ARV7^+^/K19^+^ cell [12]. Moreover, detection of AR and AR-V7 was also performed by Nimir et al. using a highly sensitive droplet digital PCR-based assay [13]. In that case, AR and AR-V7 RNA were detectable in CTCs, ctRNA and exosome samples. They could show that AR-V7 detected from CTCs could be done with a higher sensitivity and specificity compared to that detected from ctRNA and exosomes.

A second example is PD-L1 as one immune checkpoint regulator; it has become an exciting new therapeutic target leading to long lasting remissions in patients with advanced malignancies [14,15]. Kloten et al. highlighted the use of CTCs as a complementary diagnostic tool for PD-L1 expression analysis in advanced NSCLC patients [16].

Concerning (i) the stemness status of cancer cells, Strati et al. showed that the detection of TWIST1 overexpression and stem-cell (CD24, CD44, ALDH1) transcripts in EpCAM^+^ CTCs provides prognostic information in early-stage breast cancer patients [17]; and (ii) the capacity to disseminate, Huaman et al. studied hepatocellular carcinoma and castration-resistant prostate cancer and showed that CTCs exhibit distinct characteristics from primary tumor-derived cells: they highlighted an enhanced migration in part through fibronectin regulation of integrin B1 and SLUG [18].

While CTCs have long been considered to be isolated cells circulating in the bloodstream, recent research demonstrated the close interaction of CTCs with the blood microenvironment. CTCs need to establish close interaction not only with platelets and neutrophils, but also with macrophages and endothelial cells to resist the physical stress in the bloodstream. Heeke et al. discussed the recent research on the crosstalk between CTCs and the blood microenvironment and outlined currently investigated treatment strategies [19] and Garrido-Navas et al. reported the findings regarding active interactions between CTCs and platelets, myeloid cells, macrophages, neutrophils, and other hematopoietic cells that aid CTCs to evade the immune system and enable metastasis [20]. In addition, Cleris et al. detected and analyzed the morphology of CTCs and could show that orthotopic xenografts of breast cancer cell lines offer valid models of hematogenous dissemination and a possible experimental setting to study CTC–blood microenvironment interactions [21]. 

As microsatellite instability (MSI) in colorectal cancer is a marker of immunogenicity and associated with an increased abundance of tumour infiltrating lymphocytes (TILS), Toh et al. compared for the first time the MSI status with the prevalence of CTCs in the peri-operative colorectal surgery setting [22].

In cancer biology, a crucial point is to identify the CTCs able to initiate metastases. Different methods have been developed to expand CTCs in vitro and in vivo with the aim of characterizing functional metastasis-initiator CTCs with stemness traits, and to obtain new diagnostics and therapeutic tools [23]. Tayoun et al. evaluated CTC-derived models generated in different types of cancer and shed a light on challenges and key findings associated with these novel assays [24].

CLINICAL STUDIES. Rossi et al. discussed how CTCs could drastically improve tumor companion diagnostics, personalized treatment strategies, overall patient’s management, and reduce healthcare costs. Broncy et al. highlighted the clinical impact of CTCs in patients with localized prostate cancer [25] and Schochter et al. summarized the completed and ongoing clinical trials using CTC number or phenotype for treatment decisions in breast cancer [26].

Concerning colorectal cancer, the management of patients and potentially resectable liver metastases requires quick assessment of mutational status and of response to pre-operative systemic therapy. In a prospective phase II trial (NCT01442935), Bidard et al. investigated the clinical validity of CTCs and ctDNA [27]. They concluded that ctDNA detection could help to select patients eligible for liver metastases resection. In addition, Troncarelli Flores et al. reported that molecular and kinetic analyses of CTCs are predictive markers of treatment response in locally advanced rectal cancer patients [28].

Finally, metastatic melanoma is one of the most aggressive and drug-resistant cancers with very poor overall survival. Circulating melanoma cells (CMCs) were first described in 1991 and here, Cayrefourcq et al. developed a new EPISPOT assay to detect viable CMCs based on their secretion of the S100 protein using the functional S100-EPISPOT assay [29]. They showed that the S100-EPISPOT sensitivity was significantly higher than that of the CellSearch^®^ system. It will be interesting in the future to determine whether this functional test could be used in patients with non-metastatic melanoma for the early detection of tumor relapse and for monitoring the treatment response. 

## Figures and Tables

**Figure 1:**
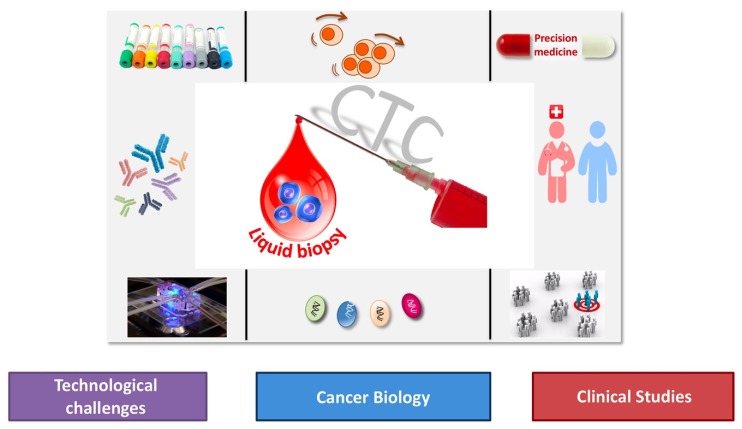
The different aspects of Liquid Biopsy: Technologies–Biology–Trials.
